# Examining Safe Food-Handling Knowledge, Behaviour, and Related Psychological Constructs among Individuals at Higher Risk of Food Poisoning and the General Population

**DOI:** 10.3390/foods12173297

**Published:** 2023-09-02

**Authors:** Jessica Charlesworth, Barbara A. Mullan

**Affiliations:** 1 enAble Institute, Faculty of Health Sciences, Curtin University, Kent Street, Bentley, WA 6102, Australia; 2 School of Population Health, Curtin University, Kent Street, Bentley, WA 6102, Australia

**Keywords:** food safety, perceived risk, habit, self-efficacy, subjective norms

## Abstract

Safe food-handling knowledge and behaviour are low across the general population. This raises concerns about whether individuals at higher risk of food poisoning have sufficient safe food-handling knowledge and engage in safe food-handling practices. The aim of this study was to explore safe food-handling knowledge, behaviour, and related psychological constructs among individuals at higher risk of food poisoning and compare the results to the general population. Participants (*N* = 169) completed measures of safe food-handling knowledge, intention, habit strength, perceived risk, self-efficacy, subjective norms, and behaviour. A series of multivariate analyses of variance were conducted to determine differences in these measures between participants at higher risk of food poisoning and the general population. No significant differences in knowledge, intention, habit strength, self-efficacy, subjective norms, and behaviour were found between individuals at higher risk of food poisoning and the general population. However, individuals at higher risk of food poisoning appeared to have stronger risk perceptions across safe food-handling behaviours compared with the general population. This study demonstrated that individuals at higher risk of food poisoning do not have higher safe food-handling knowledge than the general population, and despite having higher risk perceptions around some safe food-handling behaviours, they do not differ in engagement in safe food-handling behaviours or the majority of related psychological constructs. Implications of these findings relate to the need to target other psychological constructs, not just risk perceptions, in order to see safer food-handling behaviours in high-risk populations.

## 1. Introduction

Worldwide, one in ten people become ill with food poisoning each year [1]. While the symptoms of food poisoning can be mild, they can also be severe, leading to hospitalisation and death. It has been estimated that over 420,000 people around the world die from food poisoning each year [1]. In Australia, 4.1 million people are impacted by food poisoning each year [2], with over 30,000 hospitalisations and 80 deaths recorded each year as a result [3,4]. The impact of food poisoning extends to economic costs which exceed AUD 2.4 billion each year, resulting from productivity losses, medical care, and premature death [5].

Most cases of food poisoning occur in the home and are preventable with simple safe food-handling practices [6,7,8,9,10,11]. Guidelines for safe food handling within the home indicate that these protective behaviours include cleaning hands and surfaces before preparing food, separating raw (e.g., raw chicken) and ready-to-eat (e.g., salad) foods, cooking food thoroughly, and keeping food at the correct temperature [6,12]. However, knowledge of safe food-handling behaviours, and engagement in these behaviours, is low across the population [13,14,15,16,17].

While anyone can become ill with food poisoning, some people are at higher risk of food poisoning, including children under five years of age, pregnant people, adults over 65 years of age, and people with weakened immune systems (e.g., due to illness) [18,19,20]. These groups represent nearly 20% of the population in countries such as the United States and the United Kingdom [21], and children under five years of age carry the highest burden of food poisoning globally [1]. Further, individuals at higher risk of food poisoning underestimate their risk and tend not to worry about food safety [22]. A systematic review and meta-analysis exploring psychosocial and health-status predictors of safe food handling also found no consistent relationship between safe food handling and health-risk status [23], suggesting that being at higher risk of food poisoning may not lead to engaging in better safe food-handling practices.

Research examining food safety knowledge among individuals with compromised immune systems found that only 27% of these individuals correctly identified all risks associated with food poisoning [24]. Additionally, the mean knowledge score for safe food handling among parents of children between two and three years of age only just approached 50% [25]. Further, among caregivers of children under two years of age, barriers to handling food safely related to a lack of knowledge around specific safe food-handling practices, a lack of knowledge of the risks of some unsafe food-handling practices, and a lack of perceived importance for some safe food-handling practices [26]. This suggests that both individuals at higher risk of food poisoning and individuals who prepare food for those at higher risk may not know how to safely handle food to prevent food poisoning.

While knowledge is an important influence on some safe food-handling behaviours [25], other psychological constructs may also be important. A recent systematic review identified a number of psychological variables that were important to safe food-handling behaviour, including intention, subjective norms (i.e., perceptions that others think the behaviour is important), self-efficacy (i.e., confidence in performing the behaviour), habit strength, and perceived risk [27]. Recent research following that review supported the finding that self-efficacy [28], subjective norms [29], and intention [30] all influenced safe food-handling behaviour. Habit strength and perceived risk are two additional variables that were implicated [31,32,33], demonstrating the importance of exploring a variety of psychological constructs when investigating safe food handling in different populations. It is important to note, however, that the safe food handling outcomes and measures in the abovementioned studies varied, and the influence of different psychological constructs varied across different safe food-handling behaviours.

While limited research has examined psychological constructs related to safe food handling among individuals at higher risk of food poisoning, these psychological constructs do appear to be important. For instance, one study among older adults [34] found that intention, perceived behavioural control (similar to self-efficacy), subjective norm, and habit strength were all important for safe food handling among individuals at higher risk of food poisoning. Additional survey and mixed-methods research found that pregnant people and parents with young children had high confidence (self-efficacy) in performing safe food-handling practices [35,36]. However, there were also gaps in safe food-handling knowledge and skills among these individuals. A systematic review and thematic synthesis found that individuals are more likely to handle food more safely when they are preparing food for people at higher risk of food poisoning [37], indicating a potential influence of subjective norms and protection motivation. In relation to risk perceptions, another study revealed that older adults perceived themselves to have a lower risk of food poisoning compared to individuals in the general population [38], though another study among families with young children found that these individuals were aware of the increased risk when preparing food for individuals at higher risk of food poisoning [39].

The research described above indicates that psychological constructs, including intention, self-efficacy, subjective norm, perceived risk, and habit strength, are important for safe food-handling behaviour among the general population and among those at higher risk of food poisoning. However, studies vary in relation to the populations investigated, constructs measured, and safe food-handling behaviours examined. Additionally, research investigating differences in these constructs among individuals at higher risk of food poisoning and the general population has not yet been conducted.

Thus, the aim of this study was to explore safe food handling among individuals at higher risk of food poisoning, including safe food-handling knowledge, intention, habit strength, perceived risk, self-efficacy, subjective norms, and behaviour, and whether these factors are different when compared to the general population. Given the need for individuals at higher risk of food poisoning to be more vigilant of safe food handling, it was anticipated that there would be differences in knowledge, behaviour, and related psychological constructs across the two groups, such that those at higher risk of food poisoning would have higher knowledge, self-efficacy, subjective norms, and engagement in safe food-handling behaviour, as well as stronger habits and stronger risk perceptions.

## 2. Materials and Methods

### 2.1. Procedure

Prior to data collection, ethical approval was obtained from the University’s Human Research Ethics Committee. Participants were recruited online, Australia-wide, using paid Facebook advertising from December 2021 to January 2022. Respondents who were interested in participating were directed to an online survey on Qualtrics, where they were provided with an information sheet and an informed consent form to participate. Once participants provided informed consent, they completed a CAPTCHA and two attention check items to ensure bots were not able to continue completing the survey. Participants then completed the survey. One week later, participants were contacted via email to complete a follow-up survey, which contained a measure of safe food-handling behaviour. The part one survey took approximately 20 min to complete, and the part two survey took less than 5 min to complete. Participants had the option to enter a prize draw to win one of four AUD $50 shopping vouchers after completing the part two survey.

### 2.2. Measures

Four target behaviours, in line with current safe food-handling guidelines [6,12] were investigated: (1) cleaning hands and benchtops before and after preparing food, (2) keeping raw food (e.g., raw meat) separate from ready-to-eat foods (e.g., salad), (3) cooking and reheating food until it is steaming hot throughout, and (4) keeping perishable foods refrigerated or frozen. 

#### 2.2.1. Demographics and Risk of Food Poisoning

Participants were asked to indicate their gender identity, age, country of birth, and highest level of education. Participants were also asked if they were pregnant, had a compromised immune system, and were over 65 years of age, and if they prepared food for anyone who is pregnant, has a compromised immune system, is over 65 years of age, or for children under five years of age. Participants who indicated yes to any of these items were coded as 1 (at higher of food poisoning), while those who did not answer yes to any of these items were coded as 0 (not at higher risk of food poisoning).

#### 2.2.2. Safe Food-Handling Knowledge

A safe food-handling knowledge questionnaire [40] was used to assess safe food-handling knowledge. The questionnaire consisted of 28 multiple-choice items assessing knowledge about a range of safe food-handling behaviours (e.g., “What is the safest way to thaw chicken before cooking it?” and “Which is true about reheating leftover food?”). Participants could select one correct answer from four possible response options for each question and were awarded one point for each correct response. A total safe food-handling score was created by summing each correct response (possible range = 0 to 28), with higher scores indicating greater knowledge.

#### 2.2.3. Safe Food-Handling Intention

One item was used to assess the intention to engage in each of the four behaviours. The stem “Over the next week, I will try to…” preceded the four behaviours. Participants used a seven-point Likert scale ranging from 1 (strongly disagree) to 7 (strongly agree) to indicate how much they agreed. Higher scores indicated a stronger intention to engage in the behaviours.

#### 2.2.4. Safe Food-Handling Habit Strength

The self-report behavioural automaticity index [41,42] was used to assess habit strength for each target safe food-handling behaviour. The behaviour formed the stem of each item, which was followed by four habit strength statements (e.g., “I do automatically”, “I do without thinking”). Participants used a seven-point Likert scale ranging from 1 (strongly disagree) to 7 (strongly agree) to indicate how much they agreed. A mean habit strength score was created for each behaviour, whereby higher scores indicated greater habit strength.

#### 2.2.5. Safe Food-Handling Perceived Risk (In Relation to One’s Self)

Three perceived risk items were used to assess perceived risk in relation to one’s self. Two items measured perceived vulnerability to getting food poisoning if each of the target behaviours was not performed, i.e., “How likely is it that you will get food poisoning if you do not…” and “Compared to someone else of your age and gender, what is your chance of getting food poisoning if you do not...” preceded the four behaviours. Each item was rated on a five-point Likert scale ranging from 1 (extremely unlikely) to 5 (extremely likely). One additional item assessed the perceived severity of food poisoning in relation to the self (i.e., “How severe (serious) would getting food poisoning be for you?). The perceived severity item was rated on a five-point Likert scale ranging from 1 (not at all severe) to 5 (extremely severe). A mean score was created for perceived risk. Higher scores indicated greater perceived risk.

#### 2.2.6. Safe Food-Handling Perceived Risk (In Relation to Others)

Two similar items were used to assess perceived risk in relation to others. One item assessed perceived vulnerability and the other item assessed perceived severity in relation to others. A mean score was created from the two items. Higher scores indicated greater perceived risk.

#### 2.2.7. Safe Food-Handling Self-Efficacy

Guidelines set out by Schwarzer [43] were used to measure self-efficacy for each behaviour. Two statements, i.e., “I am confident that I can...” and “If I wanted to, I could…” preceded each of the behaviours. A seven-point Likert scale ranging from 1 (strongly disagree) to 7 (strongly agree) was used to assess participants’ agreeance with each item. A mean of the two items for each behaviour was created to indicate a self-efficacy score for each behaviour, with higher scores indicating higher self-efficacy.

#### 2.2.8. Safe Food-Handling Subjective Norm

Two items were used to measure subjective norm, with one item assessing injunctive norm and the other assessing descriptive norm, as per the theory of planned behaviour [44]. The injunctive norm item “The people who are important to me think I should…” and the descriptive norm item “People who are similar to me…” preceded each behaviour. Participants used a seven-point Likert scale ranging from 1 (strongly disagree) to 7 (strongly agree) to respond to the items. A mean subjective norm score was created for each behaviour, with higher scores indicating higher subjective norm.

#### 2.2.9. Safe Food-Handling Behaviour

Behaviour was assessed prospectively in the part-two survey, with one item per behaviour. The statement “Over the past week, how often did you...” preceded each behaviour. Participants used a five-point Likert scale ranging from 0 (never) to (4) always, where higher values indicated more frequent engagement in each behaviour. 

### 2.3. Data Analysis

IBM SPSS Statistics v27 was used for data analysis. To determine differences in knowledge scores among participants who were at higher risk of food poisoning and the general population, an independent samples *t*-test was conducted. Next, a series of multivariate analyses of variance (MANOVA) were run to determine whether there were any differences in intention, habit strength, perceived risk (self), perceived risk (other), self-efficacy, subjective norms, and behaviour between high-risk participants and the general population. Prior to running the series of MANOVAs, assumption checks were performed by running relevant tests. Appropriate steps were taken where violations to assumptions were found, for instance, Pilla’s trace test was interpreted instead of Wilks’ lambda when Box’s test of equality of covariance was violated (i.e., *p* < 0.001). Additionally, where Levene’s test of equality of error variances was violated (i.e., *p* < 0.05), stricter significance was applied (*p* < 0.01 rather than *p* < 0.05) when interpreting the significance values for that particular variable [45]. However, given that the number of participants in each group (at higher risk of food poisoning and the general population) was above 30, any violations of these assumptions were less likely to matter.

## 3. Results

### 3.1. Participants

The final sample included 169 participants aged between 19 and 77 (M_age_ = 50.26 years, SD = 13.84), of whom 92% identified as female. Among the sample, 49% (*n* = 82) of the participants were at higher risk of food poisoning. Most participants were born in Australia/New Zealand (79%) and the United Kingdom/Europe (10%), and 62% had a bachelor’s degree or higher. Table 1 shows the participant characteristics.

### 3.2. Descriptive Statistics

The means and standard deviations for scores of knowledge, intention, habit strength, perceived risk (self), perceived risk (others), self-efficacy, subjective norms, and behaviour among high-risk participants, participants in the general population, and all participants in the study are shown in Table 2.

### 3.3. Differences in Safe Food-Handling Knowledge

Results of an independent samples t-test exploring differences in knowledge between high-risk participants and participants in the general population showed there were no significant differences between the two groups (*t*(167) = 0.89, *p* = 0.93, two-tailed). The degree of difference between the means for each group (mean difference = 0.47, 95% CI [−0.57,1.50]) was very small (partial eta squared (η_p_^2^) = 0.005).

### 3.4. Differences in Psychological Constructs Related to Safe Food Handling

The series of one-way between-groups multivariate analyses of variance determining differences in psychological constructs related to safe food handling between high-risk participants and participants in the general population showed no significant differences in safe food-handling intention, habit strength, self-efficacy, or subjective norm (see Table 3).

However, there was a significant difference in perceived risk (self) (Pillai’s trace (Λ) = 0.07, *F*(4, 164) = 2.93, *p* < 0.05, partial eta squared (η_p_^2^) = 0.07) whereby, when considering all safe food-handling behaviours at once, high-risk participants had higher perceived risk (self). Inspection of the dependent variables separately also revealed perceived risk (self) for cooking and reheating food until it is steaming hot throughout to be significant (*F*(1, 169) = 6.08, *p* < 0.02, η_p_^2^ = 0.04), with high-risk participants showing stronger risk perceptions (self) (*M* = 3.92, *SD* = 0.66) for cooking and reheating food until it is steaming hot throughout than participants in the general population (*M* = 3.66, *SD* = 0.73). 

Additionally, there was a significant difference in perceived risk (others) (Wilk’s Lambda (Λ) = 0.92, *F*(4, 164) = 3.66, *p* < 0.01, η_p_^2^ = 0.08), whereby, when considering all safe food-handling behaviours at once, high-risk participants had higher perceived risk (others). However, when considered separately, none of the dependent variables were significantly different between high-risk participants and participants in the general population (see Table 3).

### 3.5. Differences in Safe Food-Handling Behaviour

There were no significant differences in safe food-handling behaviour between the two groups. Table 3 shows the results of the one-way between-groups MANOVA examining differences in safe food-handling behaviours.

## 4. Discussion

The above results show that there were no significant differences in safe food-handling knowledge, intention, habit strength, self-efficacy, and subjective norms between high-risk individuals and the general population. However, there were significant differences between high-risk participants and participants in the general population in relation to perceived risk, whereby participants at higher risk of food poisoning had higher risk perception scores when all behaviours were considered together. Additionally, participants at higher risk of food poisoning had higher perceived risk in relation to their own chances of getting food poisoning if they did not reheat food until it was steaming hot. This is inconsistent with prior research indicating that individuals at higher risk of food poisoning did not perceive their risk to be higher than the general population [38]. One explanation for this may be the differences in populations investigated in the current study compared with prior research and differences in methodologies. While there was a statistically significant difference in risk perceptions between those at higher risk of food poisoning and the general population, risk perceptions were only moderate across both groups. This is consistent with previous research indicating that individuals at higher risk of food poisoning are not aware of their susceptibility and the increased severity of food poisoning to them [46,47]. Given that there is limited literature exploring the differences in risk perceptions among high-risk individuals and the general population, these findings indicate a need to further investigate risk perceptions among these groups in order to better understand this key psychological construct for preventing food poisoning.

It is important to note that while there were no statistically significant differences between high-risk participants and participants in the general population across most variables of interest, there was room for improvement in many variables of interest. For example, the mean score for safe food-handling knowledge was only 65% across both groups, comparable to other studies where the mean safe food-handling knowledge was 60% [40] and 47% [14] among the general population. In relation to high-risk populations, recent research showed that there were gaps in knowledge about safe food-handling practices [48,49]. Taken together, these findings indicate that further work is needed to improve knowledge and risk perceptions around safe food handling among both individuals at higher risk of food poisoning and the general population.

Knowledge can be improved among individuals at higher risk of food poisoning (pregnant people and people with diabetes) using a positive deviance approach (i.e., discussing food safety behaviours and deciding and recommending more positive practices) [50]. This modelling and problem-solving approach may be one avenue for improving safe food-handling knowledge among individuals at higher risk of food poisoning. Additionally, among pregnant people, pathogen-specific information-based interventions were shown to be more effective for knowledge and behaviour change than a general information intervention [51]. Consequently, interventions with specific food safety information may be more effective for improving safe food-handling knowledge and behaviour change among individuals at higher risk of food poisoning than interventions using a more general approach.

However, information-based interventions show mixed findings in relation to improving safe food-handling knowledge. For instance, an evaluation of a pilot safe food-handling media campaign found that the information-based messages did not improve safe food-handling knowledge immediately following the campaign [52], while an evaluation of the long-term effects of the full launch of the campaign found that knowledge improved two-months following the campaign [17]. Research delving further into the mechanisms of change for the campaign found that perceived risk was an important factor for improving safe food-handling behaviour [53]. These findings are consistent with research indicating that behaviour change techniques targeting perceived risk were important for improving both perceived risk and safe food-handling behaviours [33]. However, that research was conducted among the general population, and thus, further research is needed to determine if information-based and risk-based interventions are effective for improving knowledge and perceived risk among individuals at higher risk of food poisoning.

The current study showed that both individuals at higher risk of food poisoning and the general population had high levels of intention, habit strength, subjective norm, and self-efficacy across all safe food-handling behaviours, and both groups had high levels of engagement in each safe food-handling behaviour examined. This is consistent with prior research suggesting that individuals at higher risk, and who prepare food for those at higher risk, score highly on these constructs [25,34]. However, high engagement in safe food-handling behaviour is inconsistent with research indicating that engagement in safe food-handling behaviour among individuals at higher risk of food poisoning is not sufficient to protect themselves from food poisoning [54]. This is interesting given that participants scored highly on most other constructs, yet knowledge remained low. However, this is not uncommon in the safe food-handling domain, where engagement in behaviour is high while knowledge remains low [25,55]. It may be that more specific behaviours need to be examined (e.g., cooking specific foods until cooked rather than cooking food in general), as research has suggested the importance of looking at behaviours more specifically rather than as one unitary construct [30,56]. Future research examining more specific safe food-handling behaviours, perhaps using observation, may provide further insight into this discrepancy in findings.

### Strengths, Limitations, and Future Directions

The key strength of the current study is that it contributes important insight into the safe food-handling domain in an area of research that requires further attention. The methodology used in this study, whereby scores on a variety of psychological constructs related to safe food handling were explored among both high-risk participants and participants in the general population, allowed for further insight into the need for interventions targeting knowledge and risk perceptions for those at higher risk. Further work is needed to better understand how to improve risk perception and knowledge for more specific safe food-handling behaviours, perhaps using behaviour change techniques targeting risk perception that have shown to be effective among the general population [33].

One limitation of the current study was that some specific, more risky, unsafe food-handling behaviours (e.g., cooking and handling raw eggs and raw chicken) were not examined as part of the study. As individuals at higher risk of food poisoning are not following recommendations in relation to cooking and chilling behaviours, and are also consuming high-risk foods [46,57], future research can examine more specific high-risk behaviours among individuals at higher risk of food poisoning to determine where and how to improve them. Recent research examining safe egg-handling behaviours, for instance, found that a variety of behaviour change techniques were useful for improving knowledge and perceived risk for these specific safe food-handling behaviours [30]. As this research focussed on the general population, future work examining a similar intervention among individuals at higher risk of food poisoning is needed to determine if this is effective across populations for these, and other, more specific safe food-handling behaviours. Additionally, the current study was not powered to investigate demographic differences among the variables of interest. Future research can explore this further among a larger sample of participants. Finally, the current study used only social media as a platform for recruiting participants. Future research would benefit from using social media for recruitment alongside other recruitment strategies (e.g., face-to-face recruitment, telephone recruitment) to reach a broader sample who may not use social media.

## 5. Conclusions

The findings of the current study indicate that there were no significant differences in safe food-handling knowledge, intention, habit strength, self-efficacy, or subjective norms between individuals at higher risk of food poisoning and the general population. While individuals at higher risk of food poisoning had stronger risk perceptions, the findings also indicated room for improvement in this construct, as well as safe food-handling knowledge, among both those at higher risk of food poisoning and the general population. Further research is needed to better understand where and how to improve safe food handling among individuals at higher risk of food poisoning as well as the general population.

## Figures and Tables

**Table 1:** Participant characteristics.

	*n*	%
Gender identity		
Female	13	91.7
Male	155	7.7
At-risk status		
At higher risk of food poisoning	82	48.5
General population	87	51.5
Country of birth		
Asia	12	7.1
Australia/New Zealand	133	78.7
South Africa	2	1.2
United Kingdom/Europe	16	9.5
United States of America/Canada	4	2.4
Highest level of education		
Less than year 12	10	5.9
High school certificate	10	5.9
Diploma/TAFE	44	26.0
Bachelor’s degree	61	36.1
Postgraduate degree	43	25.4

**Table 2:** Means and standard deviations for knowledge, intention, habit, perceived risk (in relation to the self), perceived risk (in relation to others), self-efficacy, subjective norms, and behaviour among participants who were at higher risk of food poisoning and participants who were not.

	At Higher Risk	General Population	Total
	Mean ± SD		
**Knowledge ^**	18.10 ± 3.43	18.56 ± 3.36	18.34 ± 3.39
**Intention**			
Cleaning hands and surfaces	6.73 ± 0.50	6.63 ± 0.59	6.68 ± 0.55
Separating raw and ready-to-eat foods	6.84 ± 0.40	6.72 ± 0.56	6.78 ± 0.50
Cooking and heating food until steaming hot	6.79 ± 0.44	6.70 ± 0.55	6.74 ± 0.50
Keeping perishable foods refrigerated or frozen	6.83 ± 0.38	6.75 ± 0.51	6.79 ± 0.45
**Habit strength**			
Cleaning hands and surfaces	6.42 ± 0.79	6.07 ±1.17	6.24 ± 1.02
Separating raw and ready-to-eat foods	6.49 ± 0.87	6.26 ±1.24	6.38 ± 1.08
Cooking and heating food until steaming hot	6.32 ± 1.00	6.12 ± 1.18	6.22 ± 1.10
Keeping perishable foods refrigerated or frozen	6.66 ± 0.57	6.58 ± 0.73	6.62 ± 0.65
**Perceived risk—of self**			
Cleaning hands and surfaces	3.78 ± 0.61	3.59 ± 0.65	3.68 ± 0.64
Separating raw and ready-to-eat foods	3.98 ± 0.63	3.74 ± 0.75	3.86 ± 0.70
Cooking and heating food until steaming hot	3.92 ± 0.66	3.66 ± 0.73	3.79 ± 0.71
Keeping perishable foods refrigerated or frozen	3.99 ± 0.65	3.87 ± 0.77	3.93 ± 0.72
**Perceived risk—of others**			
Cleaning hands and surfaces	3.72 ± 0.65	3.53 ± 0.75	3.62 ± 0.71
Separating raw and ready-to-eat foods	3.82 ± 0.66	3.64 ± 0.75	3.73 ± 0.71
Cooking and heating food until steaming hot	3.80 ± 0.66	3.56 ± 0.73	3.67 ± 0.71
Keeping perishable foods refrigerated or frozen	3.78 ± 0.64	3.71 ± 0.75	3.74 ± 0.70
**Self-efficacy**			
Cleaning hands and surfaces	6.84 ± 0.37	6.85 ± 0.32	6.85 ± 0.34
Separating raw and ready-to-eat foods	6.85 ± 0.33	6.89 ± 0.31	6.87 ± 0.32
Cooking and heating food until steaming hot	6.78 ± 0.42	6.85 ± 0.34	6.82 ± 0.38
Keeping perishable foods refrigerated or frozen	6.83 ± 0.36	6.88 ± 0.34	6.86 ± 0.35
**Subjective norms**			
Cleaning hands and surfaces	6.06 ± 0.97	6.16 ± 0.92	6.11 ± 0.94
Separating raw and ready-to-eat foods	6.14 ± 0.87	6.23 ± 0.85	6.19 ± 0.86
Cooking and heating food until steaming hot	6.15 ± 0.88	6.05 ± 0.98	6.09 ± 0.93
Keeping perishable foods refrigerated or frozen	6.29 ± 0.84	6.39 ± 0.83	6.34 ± 0.84
**Behaviour**			
Cleaning hands and surfaces	3.56 ± 0.64	3.60 ± 0.64	3.58 ± 0.64
Separating raw and ready-to-eat foods	3.87 ± 0.44	3.87 ± 0.44	3.87 ± 0.39
Cooking and heating food until steaming hot	3.84 ± 0.37	3.76 ± 0.45	3.80 ± 0.42
Keeping perishable foods refrigerated or frozen	3.87 ± 0.34	3.91 ± 0.29	3.89 ± 0.31

Note. SD = standard deviation; ^ knowledge is not specific to each behaviour.

**Table 3:** Results from the series of one-way between-subjects multivariate analyses of variance examining differences in safe food-handling intention, habit, perceived risk (in relation to the self), perceived risk (in relation to others), self-efficacy, subjective norms, and behaviour between participants who were at higher risk of food poisoning and participants who were not.

	*F*	*df*	*p*	*η_p_* ^2^
**Intention**				
Cleaning hands and surfaces	1.28	1	0.26	0.01
Separating raw and ready-to-eat foods	2.30	1	0.13	0.01
Cooking and heating food until steaming hot	1.32	1	0.25	0.01
Keeping perishable foods refrigerated or frozen	1.31	1	0.25	0.01
**Habit strength**				
Cleaning hands and surfaces	5.20	1	0.02	0.03
Separating raw and ready-to-eat foods	1.93	1	0.17	0.01
Cooking and heating food until steaming hot	1.32	1	0.25	0.01
Keeping perishable foods refrigerated or frozen	0.60	1	0.44	0.00
**Perceived risk—of self**				
Cleaning hands and surfaces	3.68	1	0.06	0.02
Separating raw and ready-to-eat foods	5.24	1	0.02 ^	0.03
Cooking and heating food until steaming hot	6.08	1	0.02 *	0.04
Keeping perishable foods refrigerated or frozen	1.15	1	0.29	0.01
**Perceived risk—of others**				
Cleaning hands and surfaces	3.12	1	0.08	0.02
Separating raw and ready-to-eat foods	2.53	1	0.11	0.02
Cooking and heating food until steaming hot	5.03	1	0.03 ^	0.03
Keeping perishable foods refrigerated or frozen	0.47	1	0.49	0.00
**Self-efficacy**				
Cleaning hands and surfaces	0.03	1	0.86	0.00
Separating raw and ready-to-eat foods	0.57	1	0.45	0.00
Cooking and heating food until steaming hot	1.44	1	0.23	0.01
Keeping perishable foods refrigerated or frozen	0.86	1	0.36	0.01
**Subjective norms**				
Cleaning hands and surfaces	0.42	1	0.52	0.00
Separating raw and ready-to-eat foods	0.46	1	0.50	0.00
Cooking and heating food until steaming hot	0.49	1	0.49	0.00
Keeping perishable foods refrigerated or frozen	0.59	1	0.45	0.00
**Behaviour**				
Cleaning hands and surfaces	0.19	1	0.67	0.00
Separating raw and ready-to-eat foods	0.00	1	0.96	0.00
Cooking and heating food until steaming hot	1.18	1	0.28	0.01
Keeping perishable foods refrigerated or frozen	0.44	1	0.51	0.00

Note. *η_p_*^2^ = partial eta squared; ^ no longer significant due to application of stricter significance in response to Bonferroni correction and/or violations to Levene’s test of equality of error variances; * significant result.

## Data Availability

The data presented in this study are available on request from the corresponding author. The data are not publicly available due to ethics protocol.
